# Exogenous serpin B1 restricts immune complex-mediated NET formation *via* inhibition of a chymotrypsin-like protease and enhances microbial phagocytosis

**DOI:** 10.1016/j.jbc.2024.107533

**Published:** 2024-07-04

**Authors:** Ting Wang, Arpit Rathee, Philip A. Pemberton, Christian Lood

**Affiliations:** 1 Division of Rheumatology, Department of Medicine, University of Washington, Seattle, Washington, USA; 2 Redd Pharmaceuticals Inc, Belmont, California, USA

**Keywords:** serpin B1, neutrophil, autoimmunity, immune complexes, activation, NET formation

## Abstract

Immune complex (IC)-driven formation of neutrophil extracellular traps (NETs) is a major contributing factor to the pathogenesis of autoimmune diseases including systemic lupus erythematosus (SLE). Exogenous recombinant human serpin B1 (rhsB1) can regulate NET formation; however, its mechanism(s) of action is currently unknown as is its ability to regulate IC-mediated NET formation and other neutrophil effector functions. To investigate this, we engineered or post-translationally modified rhsB1 proteins that possessed specific neutrophil protease inhibitory activities and pretreated isolated neutrophils with them prior to inducing NET formation with ICs derived from patients with SLE, PMA, or the calcium ionophore A23187. Neutrophil activation and phagocytosis assays were also performed with rhsB1 pretreated and IC-activated neutrophils. rhsB1 dose-dependently inhibited NET formation by all three agents in a process dependent on its chymotrypsin-like inhibitory activity, most likely cathepsin G. Only one variant (rhsB1 C344A) increased surface levels of neutrophil adhesion/activation markers on IC-activated neutrophils and boosted intracellular ROS production. Further, rhsB1 enhanced complement-mediated neutrophil phagocytosis of opsonized bacteria but not ICs. In conclusion, we have identified a novel mechanism of action by which exogenously administered rhsB1 inhibits IC, PMA, and A2138-mediated NET formation. Cathepsin G is a well-known contributor to autoimmune disease but to our knowledge, this is the first report implicating it as a potential driver of NET formation. We identified the rhsB1 C334A variant as a candidate protein that can suppress IC-mediated NET formation, boost microbial phagocytosis, and potentially impact additional neutrophil effector functions including ROS-mediated microbial killing in phagolysosomes.

Neutrophils are essential cells of the innate immune system capable of recognizing pathogens due to a broad spectrum of pattern recognition receptors, not least a wide array of Fc gamma receptors (FcgRs), able to capture immune complexes (ICs) (1, 2). Upon activation by ICs, whether targeting pathogen and/or self-antigen as in autoimmune conditions, cross-linking of FcgRs activates neutrophil effector functions, including phagocytosis, degranulation, ROS generation, and formation of neutrophil extracellular traps (NETs), a cell death process in which nuclear and cytosolic content is extruded in a web-like conformation to trap and eliminate the pathogen (2, 3). However exaggerated neutrophil activation, and NET formation in particular, is detrimental to human health, with NETs contributing to vascular damage, thrombosis, malignancy, inflammation, organ damage, and autoimmunity (4, 5, 6, 7, 8, 9, 10, 11). Thus, it is essential to properly regulate IC-mediated neutrophil activation to ensure adequate clearance of the ICs, while preventing excessive neutrophil death and tissue damage. Consistently, specifically targeting NET formation by inhibiting peptidyl arginine deaminase activity leads to reduced organ damage in mouse models of lupus, arthritis, and cardiovascular disease (12, 13, 14).

Neutrophil serine proteases (NSPs) released during neutrophil activation play an important role in shaping the immune response by activating cytokines and matrix metalloproteases and cleaving cell surface receptors such as protease-activated receptors (PARs) to trigger receptor-mediated signaling pathways (15). For example, cathepsin G (hCG) cleaves PAR-4 on platelets leading to intravascular thrombosis, tissue infarction, and release of platelet granule contents while neutrophil elastase (hNE) can cleave cell surface receptors on innate immune cells required for effective phagocytosis and removal of pathogens, ICs, and apoptotic neutrophils (16, 17). Alpha 1-antitrypsin (Serpin A1, sA1, AAT) is the major human plasma protease inhibitor targeting NSPs. It has been tested as a potential anti-inflammatory agent for the treatment of autoimmune diseases including type 1 diabetes, rheumatoid arthritis, and systemic lupus erythematosus with positive results reported in each case (18). However, AAT does not inhibit NET formation (19, 20). Despite conflicting reports, the general consensus is that NSPs, in particular intracellular hNE, are involved in NET formation (21, 22). The current dogma is that ROS mediates the translocation of hNE and myeloperoxidase into the nucleus to facilitate the de-condensation of chromatin together with PAD4 (23, 24). While many studies have demonstrated that inhibition of hNE *in vitro* and *in vivo* prevents NET formation there are many other reports where elastase is not involved thus perhaps other proteases may contribute to NET formation in these cases (22, 25, 26, 27).

Neutrophils contain large amounts of serpin B1 (sB1) which is also a potent inhibitor of the major NSPs hNE, hPR3, and hCG, but not NSP4 (28, 29). Unlike AAT, which utilizes Met358 as the P1 residue to inhibit NSPs, sB1 utilizes two independent adjacent amino acids to inhibit proteases – Phe343 to inhibit chymotrypsin-like proteases and Cys344 to inhibit elastase-like proteases (28). Serpin B1 is a non-redundant critical survival factor for neutrophils during development that protects them against hPR3-mediated apoptosis and hCG-mediated necrosis (30, 31, 32). Neutrophils lacking sB1 are also predisposed to enhance NET formation induced by PMA, PAF, MIP-2, and LPS (19). Further, exogenous recombinant human sB1 (rhsB1) can restrict NET formation when added to wild-type neutrophils before treatment with these activating agents. However, the mechanism of action is unknown and it is unclear whether inhibition by rhsB1 is restricted to these agents or occurs broadly upon NET formation induced by other biologically relevant agents.

We have examined the effect of rhsB1 on neutrophil activation and NET formation induced by ICs and established that rhsB1 but not AAT, can prevent NET formation. Through the use of rhsB1 variants and protease-specific small molecule inhibitors we found this effect to be dependent on the chymotrypsin-like inhibitory properties of rhsB1 and the protease inhibited most likely to be hCG. We also found that an oxidation-resistant rhsB1 was more effective than wild-type rhsB1 or AAT at maintaining elevated levels of cell surface markers required for neutrophil adhesion, migration, and phagocytosis and at boosting intracellular ROS production. Both forms of rhsB1 were equally efficient at boosting phagocytosis of opsonized bacteria but neither had any impact on enhancing IC uptake.

## Results

### Purity and protease inhibitory activities of serpin B1 variants

**Figure 1:**
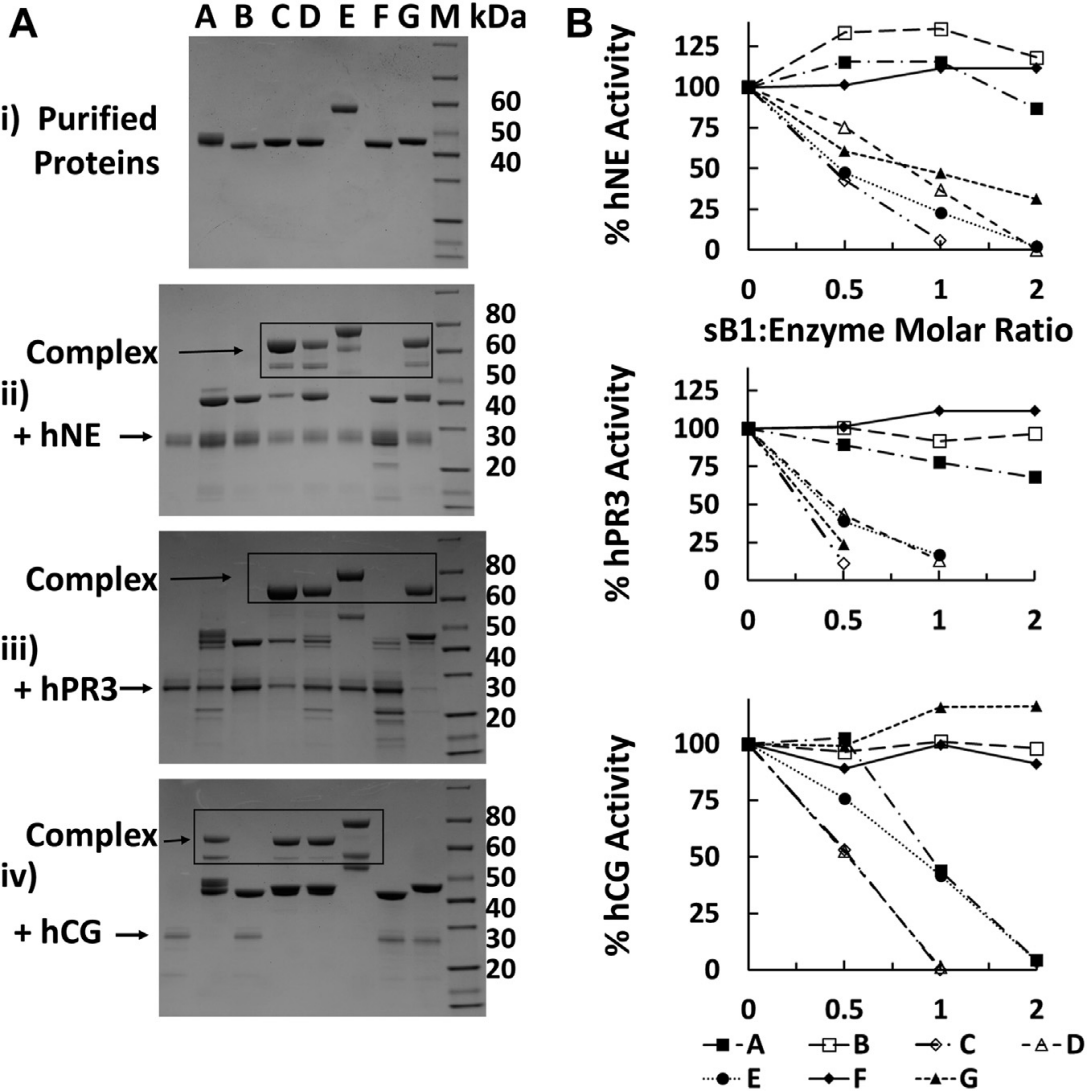
**Inhibition of NSPs by Serpin B1 variants.***A*, complex formation of sB1 variants with NSPs. *B*, inhibition of NSP amidolytic activity on chromogenic substrates by sB1 variants. Panels: (i) Purified proteins; (ii) Purified proteins + hNE; (iii) Purified proteins + hPR3; (iv) Purified proteins + hCG. M = molecular weight markers (Genscript USA Inc. Page Master protein standard plus Mol weights: 120, 80, 60, 50, 40, 30, 20, 15, and 10 kDa). For the key to *A*–*G*, please refer to Table 1.

All preparations of rhsB1 and variants thereof were >95% pure as judged by Coomassie-stained SDS-PAGE gels (Fig. 1*A* i), purified protein) and contained ≤ 0.25 EU/mg protein. We and others have previously demonstrated that wild-type rhsB1 (D) is a potent inhibitor of hNE, hPR3, and hCG but not NSP4, forming stable complexes with, and inhibiting the amidolytic activity of hNE, hPR3, and hCG at molar ratios of 2:1 or lower (Fig. 1, *A* and *B*, panels ii–iv)). The C344A variant (C) was equally potent at inhibiting hCG but more effective at inhibiting hNE and hPR3 than the wild-type protein. In contrast, and as previously described, oxidized rhsB1 (A) was unable to inhibit hNE but retained hCG inhibitory activity although it was less potent than the wild-type protein (33). In addition, we found it did not form stable complexes with hPR3 nor significantly inhibit its amidolytic activity. As predicted, reactive-site cleaved rhsB1 (B) and the T331R insertion-blocker variant (F) had no significant inhibitory activity against any of the NSPs tested. These results are summarized in Table 1.

**Table 1:** NSP inhibitory properties of serpin B1 variants

Code	Protein/Variant	Inhibitory properties	Notes
A	sB1 Ox	hNE, hPR3 (−) ∗; hCG (+) (33)	Oxidized sB1
B	sB1 RCL-cleaved	All (−)	Intact RCL required for inhibitory activity
C	sB1 C344A	All (+)	Oxidation resistant
D	sB1 C344	All (+)	Wild type
E	α_1_-antitrypsin	All (+)	Plasma-derived
F	sB1 T331R	All (−)	RCL insertion required for inhibitory activity
G	sB1 F343D, C344A, M345R	hNE, hPR3 (+); hCG (−) (36)	Selective PR3 inhibitor

### Serpin B1 inhibits IC-mediated NET formation

**Figure 2:**
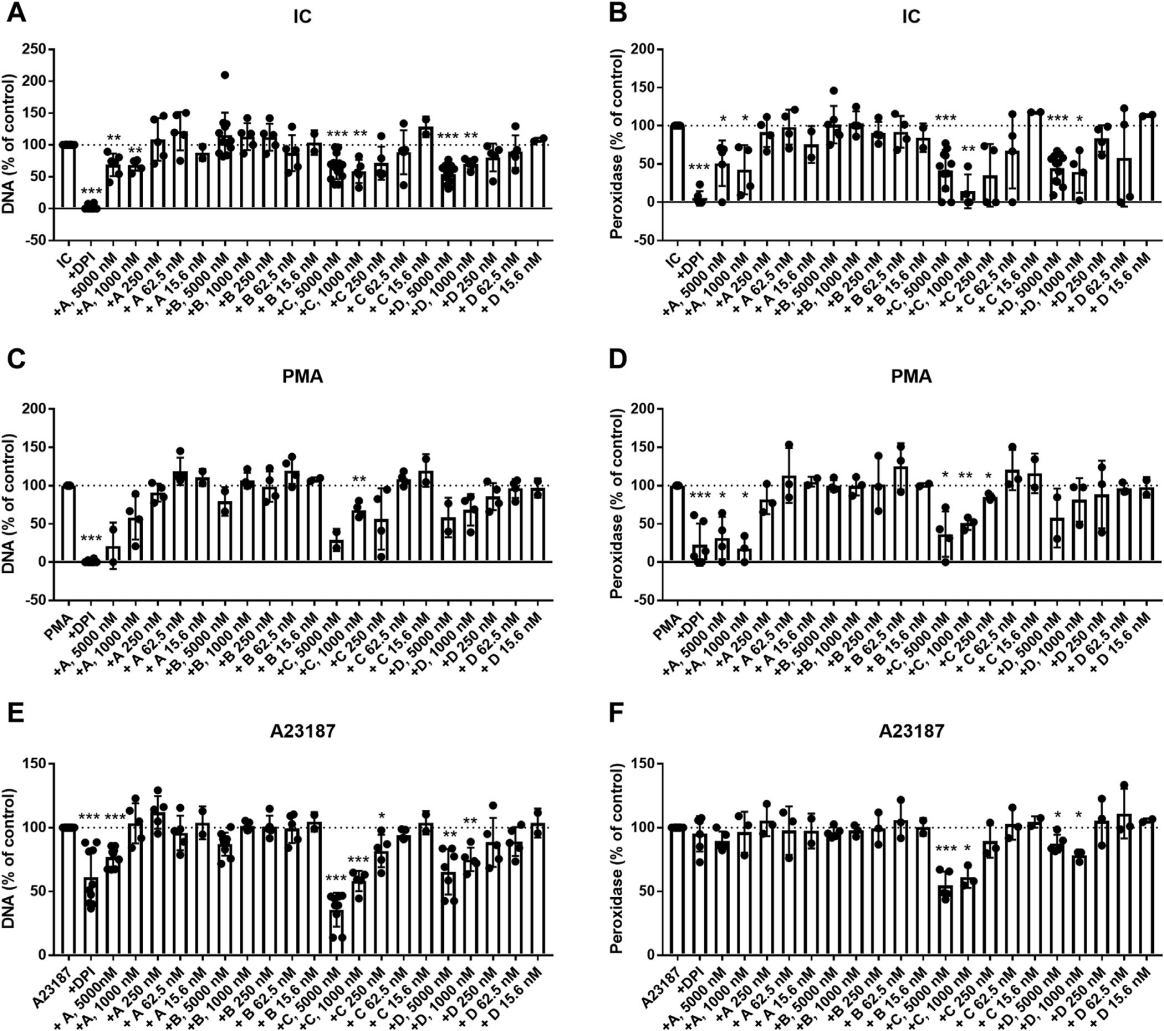
**Inhibition of NET formation by Serpin B1.** Neutrophils were pretreated with various concentrations of Serpin B1 variants and then stimulated to undergo NET formation with immune complexes (IC, panels *A* and *B*), PMA (panels *C* and *D*), or A23187. Released NETs were assessed for DNA content (*A*, *C*, and *E*) and DNA-bound peroxidase (MPO) activity (*B*, *D*, and *F*) as described in experimental procedures. Results are presented as % DNA or peroxidase activity as compared to NET-inducing stimuli alone. DPI (25 uM) was used as a positive control. The results represent the mean ± SD from more than four independent experiments. Statistical analyses were performed by paired *t* test and compared pretreated and NET agonist stimulated cells with NET agonist stimulated cells only (∗*p* < 0.05, ∗∗*p* < 0.01 and ∗∗∗*p* < 0.001).

Although rhsB1 has been previously demonstrated to block PMA, PAF, MIP-2, and LPS-mediated NET formation (19), its mechanism of action is unknown as is its ability to regulate pathophysiological relevant stimuli, including ICs which promote NET formation in autoimmune and infectious diseases. To investigate this, we used RNP-containing ICs, commonly seen in rheumatic disease, and documented NET-inducing agonists. For comparison, we used PMA to induce suicidal, NADPH-oxidase-dependent NET formation, and the calcium ionophore A23187 to induce vital, NADPH-independent NET formation (34, 35). We found wild-type rhsB1 (D), the C344A variant (C) and oxidized rhsB1 (A) to significantly and dose-dependently prevent IC-mediated NET formation (Figs. 2, *A* and *B* and S2) and consistently observed (over multiple experiments with independently isolated neutrophil preparations) ≥30 to 60% inhibition of DNA release and ≥60 to 90% inhibition of DNA associated peroxidase activity. Maximal inhibition was observed at concentrations ≥ 250nM. None of the compounds were cytotoxic (Fig. S3). Our data also demonstrated dose-dependent inhibition of PMA and A23187-mediated NET formation by all three forms of the protein, in particular using the C334A variant (D, C and A, Fig. 2, *C*–*F*). The inhibitor DPI, primarily targeting NADPH oxidase, was used as a positive control to block NET formation. Consistent with prior literature, DPI almost completely blocked PMA- and IC-mediated NET formation while only modestly reducing A23187-mediated NET formation (which is a NADPH oxidase-independent form of NET formation, Fig. 2).

### Inhibition of NET formation by sB1 requires an intact reactive center loop and the ability to undergo the S-to-R transition

**Figure 3:**
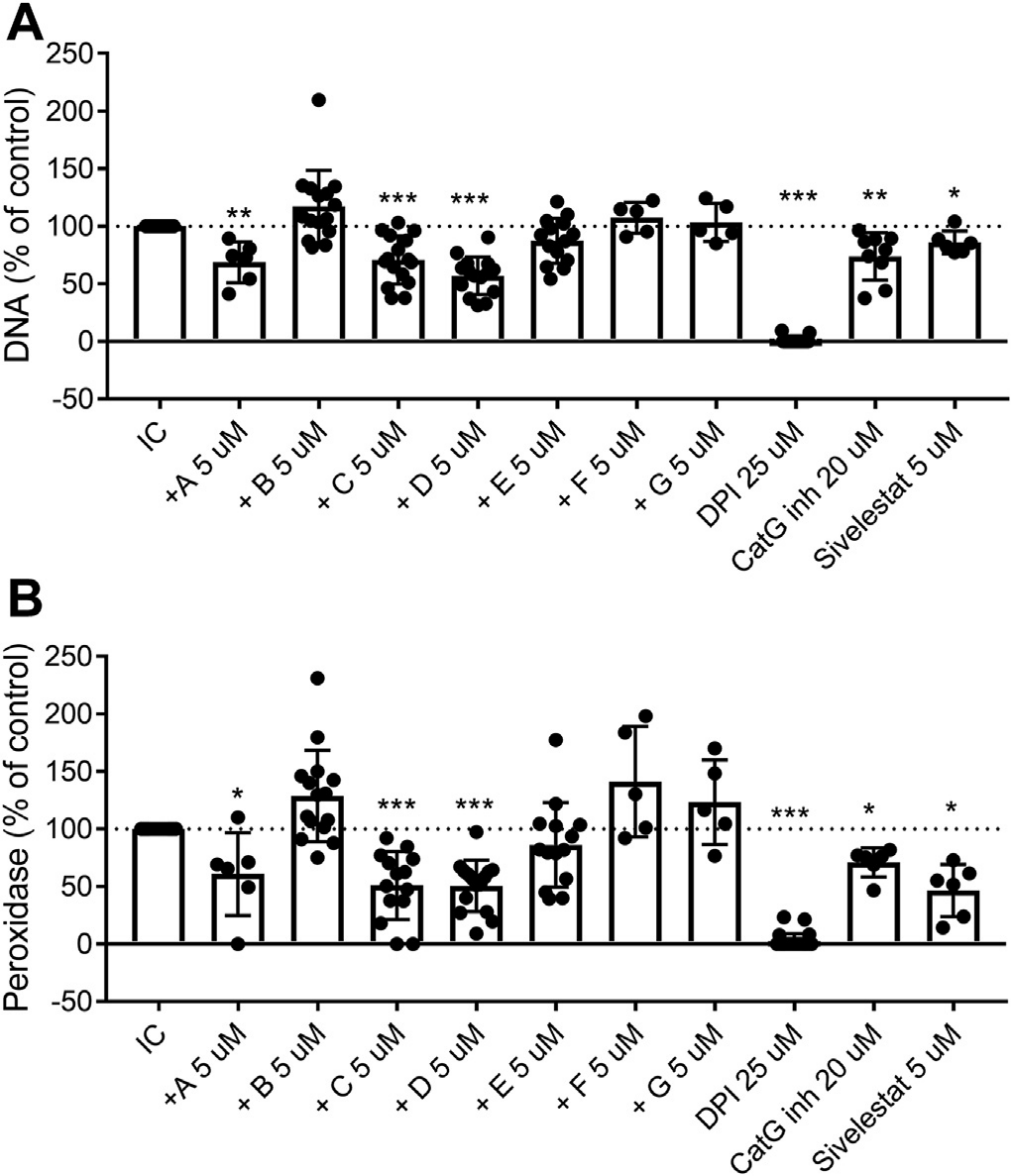
**Inhibition of IC-mediated NET formation is dependent on the chymotrypsin-like inhibitory activity of Serpin B1.** Neutrophils were pretreated with a fixed concentration of Serpin B1 variant, DPI, Cat G inhibitor or sivelestat then stimulated to undergo NET formation with IC. Released NETs were assessed for DNA content (*A*) and DNA-bound peroxidase (MPO) activity (*B*) as described in Experimental procedures. Results are presented as % DNA or peroxidase activity. The results represent the mean ± SD from more than four independent experiments. Statistical analyses were performed by paired *t* test comparing pretreated and IC stimulated cells with IC stimulated cells (∗*p* < 0.05, ∗∗*p* < 0.01 and ∗∗∗*p* < 0.001).

Next, we asked if rhsB1 inhibition of IC-mediated NET formation was dependent on having an intact RCL that could undergo the structural changes accompanying effective complex formation. Cleaving rhsB1 within the RCL induces the peptide sequence N-terminal to the reactive site to insert into β-pleated sheet A and the protein to undergo a transition from a stressed (S) to relaxed (R) state which lacks any residual protease inhibitory activity. This form of rhsB1 (B) could not prevent NET formation indicating that an intact RCL is required (Figs. 2, *A*–*F* and 3, *A* and *B*). We then asked if the conformational change that accompanies protease inhibition is required for inhibition of NET formation. For this experiment we used the rhsB1 T331R variant (F) which possesses an intact RCL but, as shown in Figure 1, cannot undergo the conformational change required for effective complex formation. This form of rhsB1 also did not inhibit NET formation indicating that the ability to undergo this conformational change is required to block NET formation (Fig. 3).

### Serpin B1 inhibition of IC-induced NET formation is dependent on its chymotrypsin-like inhibitory activity

In an attempt to define what, if any, protease(s) inhibited by rhsB1 are involved in NET formation induced by these agents we tested two variants of rhsB1 with differing protease inhibitory activities: oxidized rhsB1 (A)—which does not inhibit hNE or hPR3 but retains hCG inhibitory activity—and an engineered rhsB1 which specifically inhibits hPR3 (and hNE) but does not inhibit hCG (G). We found that oxidized rhsB1 still blocked IC-induced NET formation whereas the hPR3-specific inhibitor had no effect on NET formation induced by any of the agents tested. This suggests that rhsB1 blocks IC-mediated NET formation in a hNE- and hPR3-independent manner and that inhibition of a chymotrypsin-like enzyme, perhaps hCG, is involved.

To further examine the nature of this inhibition we tested AAT (E), the small molecule elastase inhibitor sivelestat, which is cell permeable and can inhibit NET formation *in vitro* in a hNE-dependent manner (36); a non-cell permeable specific competitive inhibitor of hCG (CatG Inh)) (37) and the NADPH oxidase inhibitor DPI. While AAT can inhibit hCG, it had no significant effect on NET formation (Fig. 3). In contrast, sivelestat had small (10–20%) but significant inhibitory effects on DNA release and peroxidase activity. CatG Inh blocked both DNA release and peroxidase activity by up to 50%—a level comparable to that routinely achieved with rhsB1 (A, C, or D) although 4-fold higher levels of the inhibitor were used (Fig. 3). DPI almost completely blocked IC- and PMA-mediated DNA release and peroxidase activity consistent with earlier reports that these are ROS-dependent events. In contrast, DPI had little effect on A23187-mediated peroxidase release but inhibited DNA release by up to 50%, consistent with earlier reports that this is a ROS-independent event (Fig. 2).

### Treatment with rhsB1 C344A amplifies neutrophil adhesion molecules and ROS formation

**Figure 4:**
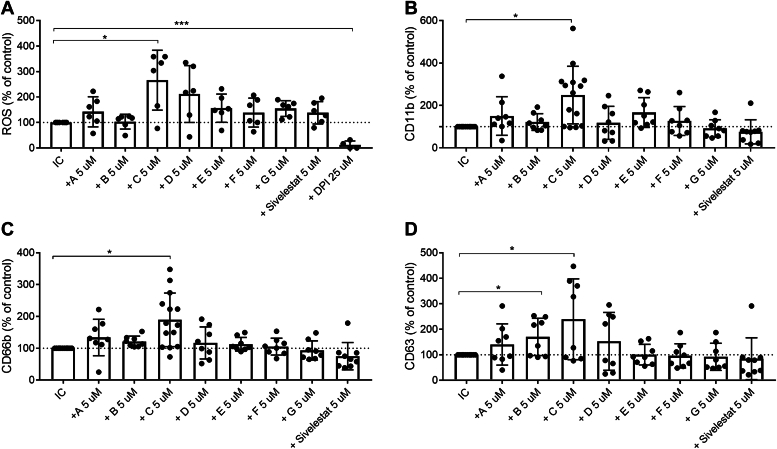
**Serpin B1 variant C334A amplifies neutrophil activation marker expression and ROS production in response to activation by ICs.** Neutrophils were pretreated with a fixed concentration of serpin B1 variant, sivelestat or DPI then stimulated to undergo NET formation with ICs. After 1 h stimulation cells were assessed for (*A*) intracellular ROS generation, (*B*) activated CD11b, (*C*) CD66b, and (*D*) CD63 cell surface levels by flow cytometry. The results represent the mean ± SD from more than four independent experiments. Statistical analyses were performed by paired *t* test comparing pretreated and IC-stimulated cells with IC-stimulated cells (∗*p* < 0.05, and ∗∗*p* < 0.01).

Given the capacity of rhsB1 and select variants to block NET formation induced by three distinct stimuli we next asked whether they altered the levels of other specific markers of neutrophil activation or impacted ROS formation. We found wild-type rhsB1 (D) (and AAT (E)) to have no significant effect on the neutrophil surface levels of activated CD11b, CD66b, or CD63 (Figs. 4, *B*–*D* and Fig. S4). It did slightly elevate ROS production; however, this result was not statistically significant (Fig. 4*A*). In contrast, the C344A variant (C) was the only agent tested that significantly elevated all three neutrophil markers by ∼200% or more and increased intracellular ROS production 2.5-fold. Sivelestat had no effect on CD11b, CD66b, CD63, or ROS production. As expected, DPI completely abolished ROS production. Thus, while NET formation was prevented by C, other neutrophil effector functions were enhanced, suggesting a skewing of the neutrophil activation response upon treatment with C but not wild-type rhsB1 or other elastase inhibitors.

### Serpin B1 enhances microbial phagocytosis but not immune complex clearance

**Figure 5:**
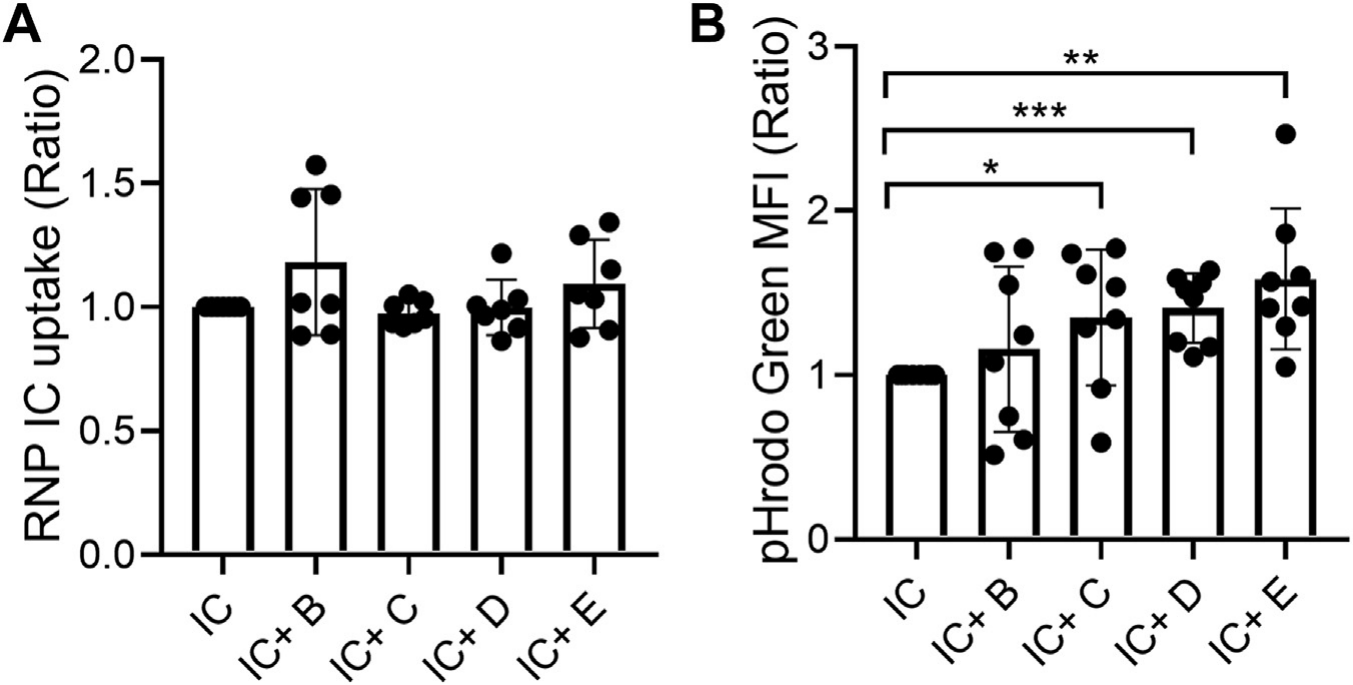
**Serpin B1 enhances microbial phagocytosis.** Neutrophils were incubated with (*A*) fluorescently labeled immune complexes, or (*B*) serum-opsonized bacteria in the presence of various Serpin B1 variants (5 uM), and assessed for uptake by flow cytometry as described in Experimental procedures. The data are represented as the ratio as compared to no Serpin B1 variant added. The results are from three independent experiments with mean ± SD. Statistical analyses were performed by paired *t* test comparing pretreated and IC-stimulated cells with IC-stimulated cells (∗*p* < 0.05, and ∗∗*p* < 0.01).

Because the rhsB1 C344A variant specifically boosted neutrophil surface levels of molecules involved in cellular adhesion, migration, and phagocytosis and prior studies suggest that sB1, like AAT, may suppress bacterial proliferation (38, 39) we next asked if rhsB1 (D) or selected variants (B and C) could impact neutrophil phagocytosis of either ICs or bacteria. We found that none of the reagents tested were able to significantly boost phagocytosis of ICs, but several were able to boost phagocytosis of complement-opsonized pHrodo Green-labeled *Staphylococcus aureus* BioParticles (Figs. 5, *A B* and S5). AAT, and rhsB1 WT (D) and C all significantly increased uptake of these particles by 20 to 30%. In contrast, the RCL-cleaved form of the protein (B) had no effect.

## Discussion

In this study, we set out to follow up on the 10-year-old observations that exogenous rhsB1 can inhibit NET formation (19) and extend those observations to include NET-formation induced by RNP-IgG ICs - the main driver of NET formation in SLE. We also sought to determine a mechanism of action by using rhsB1 variants with altered protease inhibitory activity and specificities. For the related serpin AAT, many of its immunomodulatory activities are unrelated to its ability to bind and inhibit proteases but rather depend on direct binding and sequestration of other ligands that drive inflammation within hydrophobic pockets present on the surface of the molecule (*e.g.* LTB4) (40, 41). Furthermore, AAT and sB1 both inhibit all of the major NSPs yet AAT does not inhibit NET formation whereas sB1 does, thus it is not obvious that protease inhibition would necessarily be a part of the mechanism by which rhsB1 blocks NET formation. Nevertheless, several lines of evidence point to the involvement of an NSP and in particular, hCG in mediating IC-driven NET formation in neutrophils. First, non-protease inhibitory variants of rhsB1 do not block NET formation. Second, rhsB1 variants that only inhibit hNE and PR3 also do not block NET formation whereas rhsB1 variants that specifically inhibit chymotrypsin-like proteases *via* F343 do block NET formation. Third, the concentrations and dose-dependency of rhsB1 required to inhibit NET formation are similar to the levels of intracellular NSPs available for release during neutrophil degranulation and much higher than typically required for activation of ligand-mediated receptor-mediated signaling pathways and fourth, pre-incubation with a specific hCG inhibitor also blocks NET formation. Finally, rhsB1 must be present prior to the addition of the NET-inducing agent. Adding it at the time of stimulation or later has no effect (19) suggesting that it interacts with molecules on the surface of resting neutrophils prior to neutrophil activation. Our data suggests this interaction alters how the neutrophil responds to NET-forming stimuli and that inhibition of hCG is a required part of this process.

Additional evidence supporting the involvement of hCG comes from observations made using variants of another major neutrophil protease inhibitor—secretory leukocyte protease inhibitor (SLPI). SLPI is a low molecular weight protein protease inhibitor (11.7 kDa) that also inhibits hCG and hNE, and blocks NET formation. Its mechanism of action has been partly attributed to its anti-hNE activity (42, 43). However, like rhsB1, SLPI variants lacking hNE inhibitory activity still inhibit NET formation to some extent and retain some inhibitory potential against chymotrypsin (hCG) or trypsin-like proteases. Although the definitive protease responsible for inducing NET formation was never identified in this study, the data support a role for a protease other than hNE, perhaps hCG (43). Other studies in mouse models of deep-vein thrombosis also show that elastase is not required for NET formation and subsequent production of pathological venous thrombi (26). More recently, an indirect role for hCG in activating platelet-mediated NET formation and thrombosis has emerged (44). Collectively, these findings support the involvement of NSPs other than elastase in inducing NET formation and provide evidence that hCG may play a role early on in the process of NET formation. Multiple pro-inflammatory mechanisms of action have been ascribed to hCG during inflammatory and autoimmune diseases (45, 46) and while previous studies have revealed a clear role for intracellular sB1 in preventing hPR3-mediated apoptosis and hCG-mediated necrosis, to our knowledge, this is the first study to suggest that hCG may play a role in NET formation and that exogenous sB1 may prevent that. Follow-up studies should help further elucidate hCG’s involvement in NET formation.

**Figure 6:**
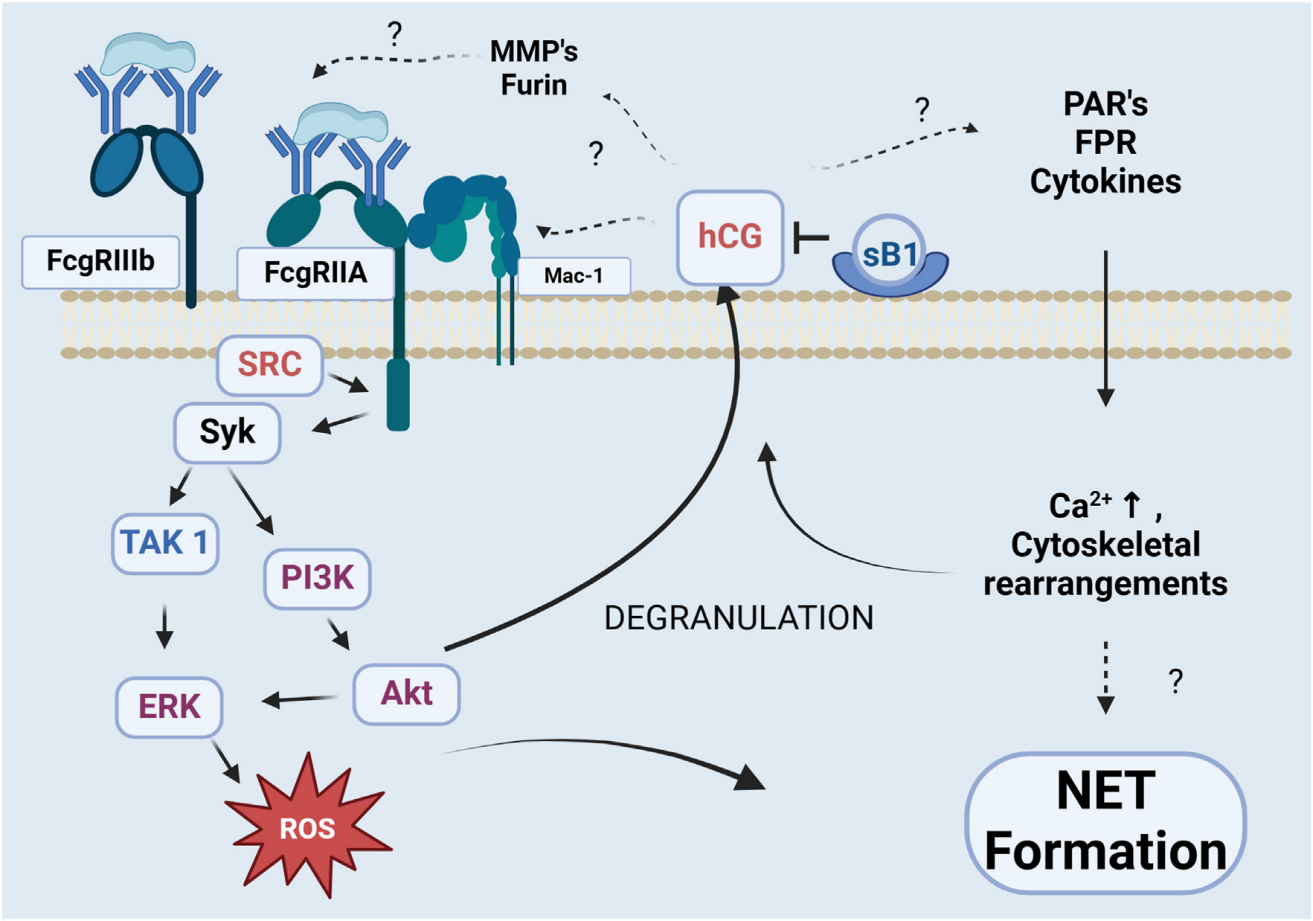
**Hypothetical role(s) of hCG in IC-mediated NET formation**. In this model, immune complexes activate members of the SRC and Syk kinase families *via* FcγRIIIB and/or FcgRIIA (70). Subsequent signaling pathways result in the rapid production of ROS and release of NSPs, including hCG, *via* degranulation. Surface-bound hCG may then induce a secondary signal required for induction of NET formation *via* one or more of the pathways indicated including integrin (Mac-1) clustering, MMP activation and subsequent Fcγ receptor shedding, and PAR, FPR, or TLR activation (*via* cytokine processing). sB1 prevents NET formation *via* a mechanism involving binding to the neutrophil cell surface and direct inhibition of hCG. Figure created with BioRender.com.

Based on these observations we propose that a chymotrypsin-like protease, most likely hCG, present on the surface of resting neutrophils and released during neutrophil activation by ICs (and other stimulants) is involved in NET formation (Fig. 6). How it is involved in NET formation is currently unclear however, it must be dependent on its proteolytic activity as both rhsB1 and the small molecule hCG inhibitor prevent NET formation. AAT also inhibits hCG yet does not inhibit NET formation induced by ICs or other activating agents. Several reasons for this may be: (1) the larger size of glycosylated AAT (54 kDa) vs non-glycosylated rhsB1 (42 kDa) sterically hindering access to hCG bound to the surface of neutrophils (47); (2) differences in the rate or stability of inhibition of hCG by the respective inhibitors. rhsB1 inhibits hCG 5-fold faster than AAT and this reaction is not markedly affected by oxidation whereas the AAT:hCG reaction rate is reduced almost 1000-fold (28, 48) or (3) Interactions of other domains on rhsB1 that are not present in AAT with cell surface receptors preventing triggering of programmed cell death pathways.

Several hCG targets on the neutrophil surface or released by neutrophils that are, or have the potential to be, involved in NET formation are the PARs (protease-activated receptors), integrins, G-protein coupled receptors, and chemokines/cytokines. For example, PAR-2 cleavage and activation by a class of bacterial-derived cysteine proteases called gingipains induces NET formation (49). In contrast, PAR-2 is also cleaved by the NSPs with varying and biased effects on PAR-2-mediated signaling (50, 51). Ramachandran *et al.* (51) found that all three NSPs disarmed PAR-2 by cleaving and releasing the N-terminal tethered ligand (TL) sequence thereby preventing signaling by other proteases. However, only elastase induced a TL-independent PAR-2 activation of MAP kinase and selective activation of the p44/42 MAPK pathway demonstrating that NSPs can signal independently to effect changes intracellularly. hCG has been shown to modulate neutrophil spreading and the clustering of integrins leading to optimal neutrophil effector functions (52). It is also a potent chemoattractant and exerts its effects by binding to formyl peptide receptor inducing a Ca2+ flux and translocation of PKCζ (53). hCG can also modulate the release and activity of chemokines and cytokines (50). Additional experiments are required to help us understand which, if any, of these pathways are affected by rhsB1 during NET formation.

Oxidation did not markedly affect the ability of wild-type rhsB1 to block NET formation or induce significant changes in levels of neutrophil activation markers or ROS production. However, an unexpected finding was that the oxidation-resistant C344A variant gave very different results. It significantly boosted levels of all three markers and ROS production while effectively suppressing NET formation. We do not currently understand how this particular variant boosts neutrophil surface levels of these markers but the increase in activated CD11b may help explain the observed increase in ROS production. NSPs and in particular hNE, are capable of degrading cell surface receptors required for efficient assembly of the NADPH oxidase (NoX) and phagocytosis including Fcγ receptors (FcγRIII), CR1 (but not CD11b/CD18 [CR3, Mac-1, α_M_β_2_]), and opsonins (iC3b) and opsonin receptors (54, 55, 56). Previous studies have shown that CD11b/CD18 works in concert with FcγRIII to generate the neutrophil respiratory burst and has a requirement for tyrosine phosphorylation on the ITAM (immunoreceptor tyrosine-based activation motif) domain of FcγRIIA and subsequent signaling (57, 58). Thus, hNE shedding of FcγRIII (or FcγRIIA) would render it unable to drive assembly of the NoX complex while inhibition of hNE by effective elastase inhibitors including the C344A variant should prevent that. However, silvelestat and AAT also had no effect on ROS production most likely because they did not boost CD11b activation and optimal ROS production necessarily requires both CD11b and FcγRIII.

Other proteases also contribute to the shedding of neutrophil cell surface receptors. The main neutrophil matrix metalloprotease responsible for shedding FcγRIIIB (CD16B) is ADAM17 (a disintegrin and metalloprotease). ADAM17 is induced by multiple stimulants including fMLP, PMA, and FcR/CR-dependent phagocytosis and its activation requires caspase eight and the production of ROS by mitochondria. Similarly, RNP-IC activation of neutrophil TLR's 7 and/or 8 induces furin-mediated shedding of FcγRIIA reducing phagocytic activity of ICs while increasing their propensity to undergo NET formation (59). We do not yet know the effect of rhsB1 on ADAM17 or furin activity but in relation to TLR 7 and/or 8 signaling, CD11b activation has been shown to reduce inflammation and autoimmunity in SLE by suppressing TLR and IFN-I signaling *via* negative regulation of the NF-κB pathway and increased degradation of MyD88 (60, 61). This provides a clear rationale for further testing of the C344A variant in cell-based and animal models of SLE, RA, and other IC-driven autoimmune diseases.

Given the role of CD11b/CD18 in phagocytosis, we hypothesized that adding rhsB1 to IC-treated neutrophils would not only prevent NET formation but may boost their uptake of RNP-ICs and/or opsonized bacterial particles. While we did not observe improved uptake of RNP-ICs we did see increased phagocytosis of complement-opsonized pHrodo-labelled *s.aureus* bioparticles by neutrophils treated with both wild-type and C344A rhsB1 and AAT but not RCL-cleaved rhsB1. This is most likely because phagocytosis of RNP-ICs is completely dependent on FcγR-mediated uptake and, as discussed earlier, NSPs, MMPs and furin may degrade FcγRs thus abolishing uptake. In contrast, NSP (in particular hNE) degradation of CR1 and opsonins (but not CD11b/CD18), would be blocked by these three proteins thus boosting phagocytosis of opsonized bacteria *via* CR1. This is supported by the observation that the marked increase in CD11b-driven by the C344A variant alone did not result in higher phagocytosis of opsonized bacteria than that achieved with wild-type rhsB1 alone.

In conclusion, we have uncovered a mechanism of action by which exogenously added rhsB1 prevents NET formation induced by multiple stimuli including RNP-IC. The mechanism is dependent upon the inhibition of a chymotrypsin-like protease, and studies with specific inhibitors indicate that hCG is the protease responsible. We also unexpectedly uncovered a potential mechanism whereby the C344A variant boosted ROS production and clearance of opsonized particles while simultaneously suppressing NET formation.

## Experimental procedures

### Design, expression, and purification of serpin B1 variants

Recombinant human serpin B1 and several engineered variants possessing altered protease inhibitory activities were produced in and purified from the yeast *S*. *cerevisiae* as previously described (Table 1) (33). Several properties of wild-type (C344, code “D”) and an oxidation-resistant variant (C344A, code “C”) of rhsB1 have been described previously (28, 33, 62). The additional variants created used either the wild-type or C344A sB1 gene expression constructs as templates for site-directed mutagenesis. All genetic manipulations and confirmation of the final construct sequences were performed at Genscript USA Inc. The variants were T331R (wild-type template, code “F”) and F343D, C344A, and M345R (C344A template, code “G”). As has been described for other serpins, the replacement of T with R at this specific site within the hinge region of a serpin is predicted to restrict reactive center loop (RCL) mobility (“insertion blocker”) and prevent effective complex formation with a target protease. This conformational change is required for effective protease inhibition and variants with this mutation are predicted to be devoid of protease inhibitory activity (63). The F343D, C344A, and M345R variants have been described elsewhere as a specific inhibitor of hPR3 that lacks any residual chymotrypsin-like inhibitory activity but retains elastase inhibitory activity (64). All preparations of variants and post-translationally modified forms of rhsB1 were tested for endotoxin content using the gel clot method (Genscript) and if necessary treated using the phase separation method to reduce endotoxin content to levels acceptable for cell based assays (65).

### Preparation of post-translationally-modified forms of sB1

Purified wild-type rhsB1 was oxidized with NCS to create rhsB1 Ox (code “A”) as previously described (33) and re-purified by chromatography on a 5 ml Hitrap QHP column cartridge. Oxidized rhsB1 cannot inhibit either neutrophil or pancreatic elastase. Instead, it is rapidly cleaved within the RCL to yield a non-inhibitory protein (rhsB1-CL, code “B”) (33). RhsB1-CL was made from rhsB1 Ox using pancreatic elastase and re-purified by anion-exchange chromatography.

### Protease inhibitory activity of serpin B1 variants

The inhibitory activity of rhsB1 and its variants was tested against the major NSPs hNE, hCG, and hPR3 (Athens Research and Technology). Proteins were analyzed for their ability to inhibit these enzymes by two methods: (A) Formation of classical SDS and heat-stable complexes and (B) titrimetric analysis of inhibition of NSP amidolytic activity against select chromogenic substrates. For (A) aliquots (2uL) of hNE, hPR3, and hCG (23.4 uM each) were mixed with each sB1 protein at a 1:1 molar ratio in a fixed volume of 20uL PBS, pH 7.4 for 15 min. Residual enzyme activity was stopped by the addition of a protease inhibitor mix (Sigma Aldrich), and samples were analyzed by SDS-PAGE after the addition of sample buffer (reducing) and heating. For (B) each sB1 protein was incubated with each enzyme at molar ratios varying from 0.5:1 to 4:1 inhibitor-to-enzyme in a 200uL volume of PBS, pH 7.4 in a microtiter plate for 5 min. The concentration of hNE was 100 nM and residual enzyme activity (Vmax) was measured *via* the release of p-nitroaniline (405 nm) from MeO-Succ AAPV-pNA (Bachem Americas). hCG was 500 nM and residual enzyme activity (Vmax) was measured *via* the release of p-nitroaniline (405 nm) from Succ AAPF-pNA (Bachem Americas). hPR3 was 200 nM and residual enzyme activity (Vmax) was measured *via* the release of p-nitroaniline (405 nm) from MeO-Succ AAPV-pNA. All synthetic substrate final concentrations were 1 mM.

### NET formation assays

Human neutrophils were isolated by Polymorphprep (Axis-Shield) as previously described (66). For purity and gating, see Fig. S1. Red blood cells were lysed with RBC lysis buffer (BioLegend). Neutrophils were resuspended in serum-free RPMI-1640 medium (Life Technologies) for *in vitro* assays. Prior observations had determined that rhsB1 only inhibited NET formation when added prior to the addition of the NET stimulating agent (19). Hence, after 20 min settling down in poly-L-lysine-coated tissue culture plates, neutrophils (10^6^ cells/ml) were pre-incubated for another 60 min in the presence of rhsB1, an rhsB1 variant or other reagent, including the elastase specific inhibitor silvelestat (5 μM, Sigma-Aldrich, St Louis, MO, CAS # 150374-95-1, product # S7198), the NADPH inhibitor diphenyleneiodonium (DPI, 25 μM, Sigma-Aldrich, CAS # 4673-26-1, product # 300260) and the cell impermeable hCG specific inhibitor (Cathepsin G Inhibitor I, 20uM, Sigma-Aldrich, CAS # 429676-93-7, product # 219372) at the indicated concentrations prior to addition of RNP-IgG immune complexes (ICs) prepared as described (59, 67) (IgG; purified from SLE patients with high titer anti-ribonucleoprotein [RNP] antibodies mixed with SmRNP [Arotec Diagnostics, Lower Hutt, Wellington, New Zealand], and used at a final concentration of 10 μg/ml). The operator was blinded to which rhsB1 variant was used. In some experiments, neutrophils were activated with PMA (20 nM) or A23187 (25 μM). After 4 h of incubation, plate-adherent NETs were washed in PBS once, and detached using micrococcal nuclease (0.3 U/ml; Thermo Fisher Scientific, Waltham, MA), diluted in nuclease buffer containing 10 mM Tris-HCl, pH 7.5, 10 mM MgCl_2_, 2 mM CaCl_2_, and 50 mM NaCl. The DNA content of detached NETs was quantified by staining with the cell impermeable nucleic acid stain Sytox Green (Life Technologies), The peroxidase (mainly MPO) activity associated with detached NETs was quantified using the peroxidase substrate tetamethylbenzidine (TMB), by plate reader (Synergy 2; BioTek).

### Neutrophil activation assays

Neutrophils were pre-incubated with rhsB1 or a variant, then activated with RNP-IC (10 μg/ml), as above, for 3 h, and analyzed for surface levels of CD66b (mAb #305106), CD11b (mAb #301406; recognizing the activated form of CD11b), and CD63 (mAb #353008; all monoclonal antibodies diluted 1/100, BioLegend) by flow cytometry. Data were analyzed by FlowJo (Tree Star) and results presented as fold activation relative to the activation achieved with RNP-IC alone.

### ROS assays

Neutrophils were isolated and suspended in RPMI before being put on ice for 30 min. Neutrophils (1 × 10^6^ cells/ml) were then transferred into a 96-well U-bottom plate in the presence of different rhsB1 variants (5 μM) or for a 1 h incubation at 37 degrees. RNP-IC (10 μg/ml) was used to stimulate neutrophils for an additional hour at 37 degrees. During the last half hour of incubation, dihydrorhodamine 123 (DHR-123, Thermo Fisher Scientific) was added to each well (0.5uM). Cells were analyzed by flow cytometry immediately for ROS production at the end of incubation.

### Neutrophil phagocytosis of fluorescence-labeled immune complexes

Purified anti-RNP SLE IgG was labeled with Alexa Fluor 488 Antibody Labeling Kit (A20181, Thermo Fisher Scientific) at appropriate concentration according to the manufacturer’s instructions prior to incubation with SmRNP to make Alexa Fluor 488 -labeled immune complexes. Isolated neutrophils (1 × 10^6^ cells/ml) were incubated with or without different rhsB1 compounds at 5 μM (B, C, D, and E) for 1 h prior to the addition of Alexa Fluor 488-labeled immune complexes for another 1 h. At the end of incubation, the neutrophils were placed on ice to stop further phagocytosis. After washing, neutrophil phagocytosis of fluorescence-labeled immune complexes was determined with flow cytometry by assessing the mean fluorescence intensity (MFI) of Alexa Fluor 488.

### Neutrophil phagocytosis of complement-opsonized *staphylococcus aureus*

For complement opsonization, pHrodo Green *S. aureus* BioParticles Conjugate (P35367, Thermo Fisher Scientific) was dissolved in 500ul PBS, mixed with 500 μl of RPMI, and opsonized with 250ul human serum from a healthy donor for 1 h at 37 °C on a thermoshaker (400 rpm). After incubation, opsonized pHrodo Green *S. aureus* BioParticles were pelleted by centrifugation and resuspended in 440 μl of RPMI.

Neutrophils were isolated from healthy subjects with Polymorphprep (Axis-Shield) density gradient as described previously (66). Isolated neutrophils (1 × 10^6^ cells/ml) were incubated with or without different rhsB1 compounds at 5 μM (B, C, D, and E) for 1 h and stimulated with RNP-IC (10 μg/ml) for another 2 h. At the end of incubation, neutrophils were pelleted by centrifugation and resuspended in 50ul RPMI/well at a concentration of 4 × 10^6^ cells/ml. Subsequently, 20 μl of complement-opsonized pHrodo Green *S. aureus* BioParticles were added to the neutrophils and incubated for another 5 min at 37 °C on a plate thermoshaker (200 rpm). The plate was then placed on ice to stop further reaction and the relative amounts of pHrodo Green *S. aureus* BioParticles taken up by phagocytosis determined by flow cytometry (Beckman Coulter). Cells were gated based on forward scatter (FSC)/side scatter (SSC) properties, and the mean fluorescence intensity (MFI) of the pHrodo was determined using FlowJo (Tree Star) (68, 69).

### Statistical analysis

Paired *t* test were used for samples assuming Gaussian distribution. For non-parametric analyses, the Mann-Whitney *U* test and Wilcoxon signed-rank test were used. All analyses were performed in GraphPad Prism (GraphPad Software Inc.), and considered statistically significant at *p* < .05.

## Data availability

Summary data are contained within the manuscript. Specific data sets will be provided upon reasonable request to the corresponding author.

## Supporting information

This article contains supporting information.

## Conflict of interest

The authors declare the following financial interests/personal relationships which may be considered as potential competing interests:

P. A. P is an employee of Redd Pharmaceuticals Inc. C. L is a scientific advisor to Redd Pharmaceuticals Inc.
