# Supplementary material for: Exogenous serpin B1 restricts immune complex-mediated NET formation via inhibition of a chymotrypsin-like protease and enhances microbial phagocytosis
Source: J Biol Chem. 2024 Jul 4;300(8):107533. doi: 10.1016/j.jbc.2024.107533 (PMC11327461; doi:10.1016/j.jbc.2024.107533)
Supplement: Supplemental data [file mmc1.docx]

**Supplemental Data**

**
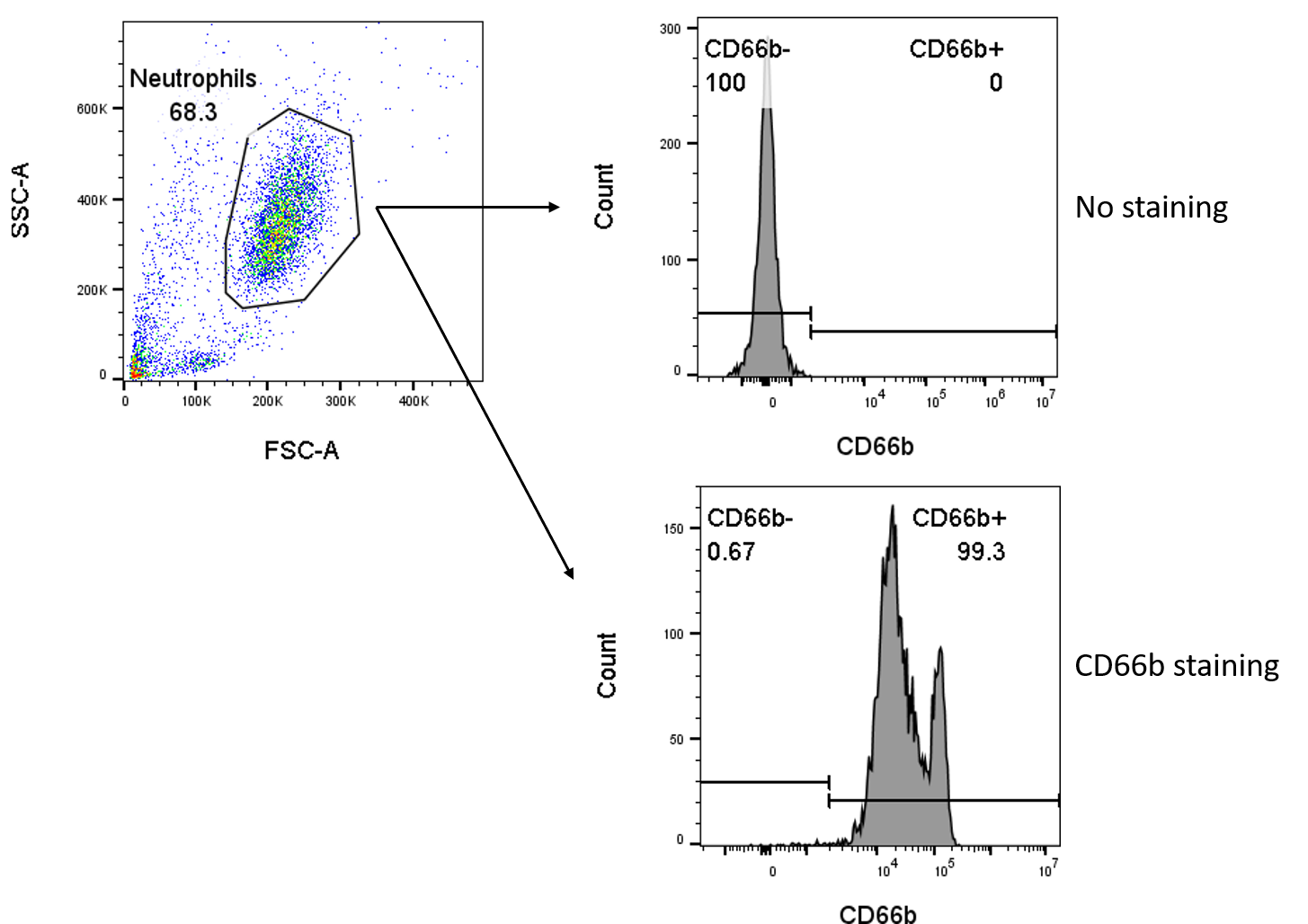
**

**Supplemental Figure 1. Neutrophil gating strategies.** Primary neutrophils were gated based on forward and side scatter properties, as well as expression of CD66b.

**
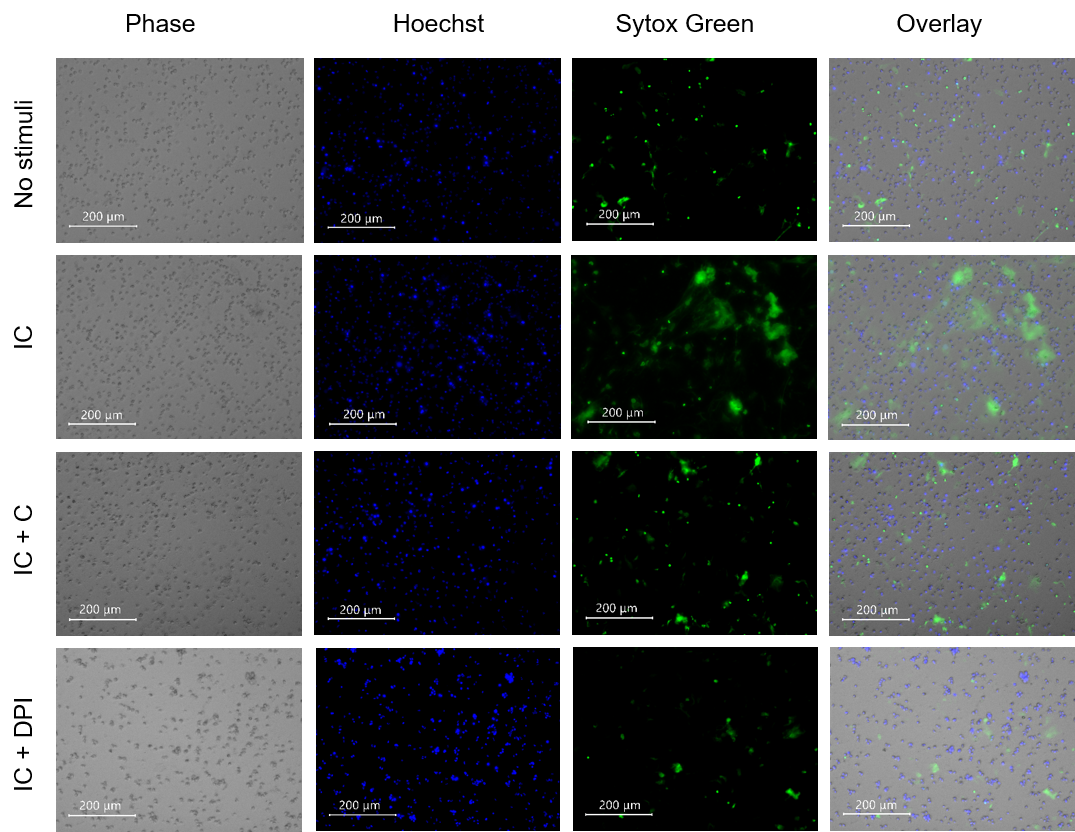
**

**Supplemental Figure 2. Neutrophil extracellular trap formation.** Neutrophil extracellular traps were visualized by microscopy using cell permeable DNA stain Hoechst (blue) and cell impermeable DNA stain Sytox Green (green). NET formation was evaluated after four hours of stimulation with immune complexes (ICs) in presence or absence of SerpinB1 compound C (5 μM) or ROS inhibitor DPI (25 μM). The scale indicates 200 μm.

**
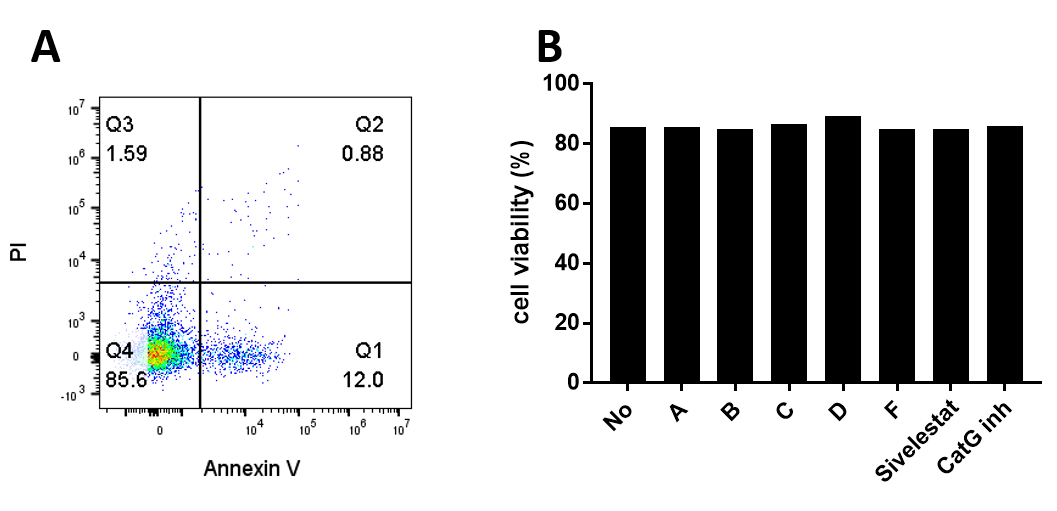
**

**Supplemental Figure 3. Neutrophil viability.** Neutrophils were incubated with indicated compound at experimental concentration and incubated for 4 hours prior to assessment of neutrophil viability as determined by propidium iodide (PI) and AnnexinV staining by flow cytometry. A) Representative gating; and B) representative quantification of cell viability from one experiment.

**
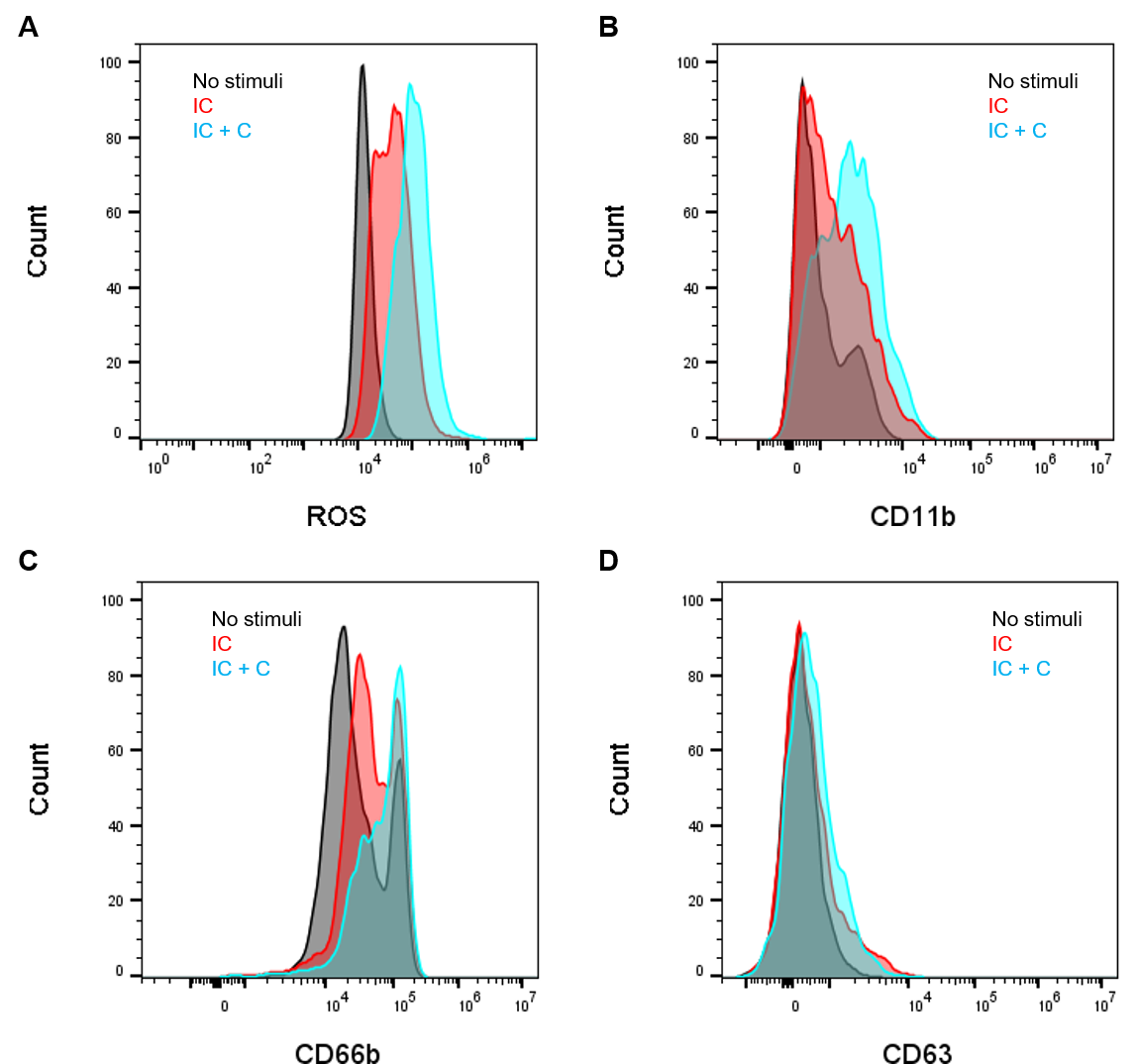
**

**Supplemental Figure 4. Neutrophil activation markers.** Representative histograms of neutrophil A) intracellular ROS generation; B) activated CD11b; C) CD66b; and D) CD63 cell surface levels. Neutrophils were incubated with (red) or without (black) immune complexes (ICs) in presence (blue) or absence (red) of SerpinB1 compound C at 5 μM.

**
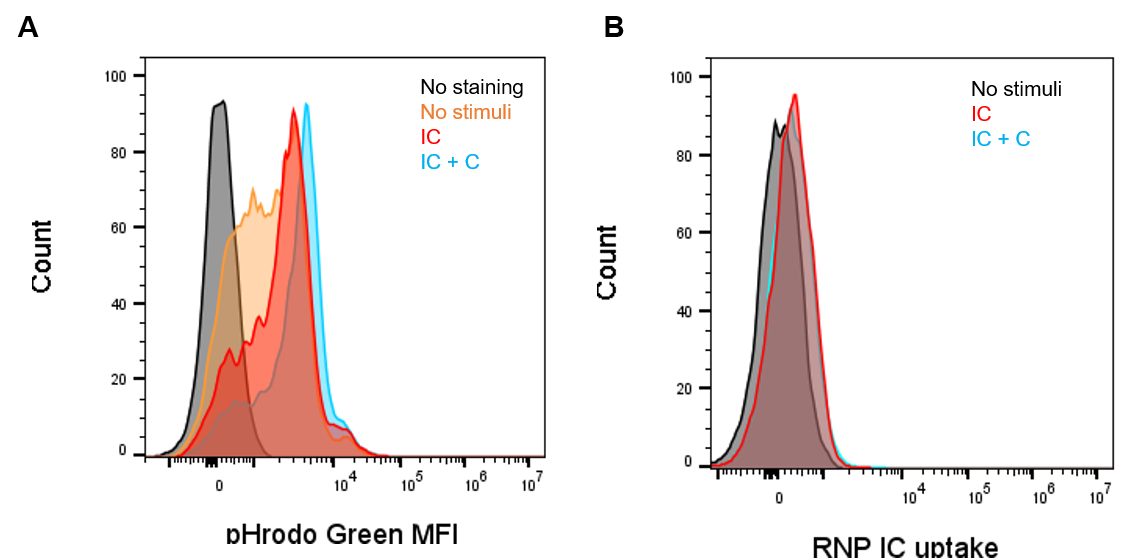
**

**Supplemental Figure 5. Neutrophil phagocytosis assays.** Representative flow cytometry histograms depicting A) uptake of serum-opsonized bacteria, and B) uptake of fluorescently labeled immune complexes (RNP IC).
